# Nuclear Multidrug-Resistance Related Protein 1 Contributes to Multidrug-Resistance of Mucoepidermoid Carcinoma Mainly via Regulating Multidrug-Resistance Protein 1: A Human Mucoepidermoid Carcinoma Cells Model and Spearman's Rank Correlation Analysis

**DOI:** 10.1371/journal.pone.0069611

**Published:** 2013-08-27

**Authors:** Bolei Cai, Ye Miao, Yuan Liu, Xiaofang Xu, Sumin Guan, Junzheng Wu, Yanpu Liu

**Affiliations:** 1 Department of Oral Biology, School of Stomatology, the Fourth Military Medical University, Xi'an, China; 2 Department of Oral and Maxillofacial Surgery, School of Stomatology, the Fourth Military Medical University, Xi'an, China; 3 Department of Preventive Dentistry and Infection Control Office, School of Stomatology, the Fourth Military Medical University, Xi'an, China; 4 Department of Oral Histology and Pathology, School of Stomatology, the Fourth Military Medical University, Xi'an, China; University of Alabama at Birmingham, United States of America

## Abstract

**Background:**

Multidrug resistance-related protein 1 (MRP1/ABCC1) and multidrug resistance protein 1 (MDR1/P-glycoprotein/ABCB1) are both membrane-bound drug transporters. In contrast to MDR1, MRP1 also transports glutathione (GSH) and drugs conjugated to GSH. Due to its extraordinary transport properties, MRP1/ABCC1 contributes to several physiological functions and pathophysiological incidents. We previously found that nuclear translocation of MRP1 contributes to multidrug-resistance (MDR) of mucoepidermoid carcinoma (MEC). The present study investigated how MRP1 contributes to MDR in the nuclei of MEC cells.

**Methods:**

Western blot and RT-PCR was carried out to investigate the change of multidrug-resistance protein 1 (MDR1) in MC3/5FU cells after MRP1 was downregulated through RNA interference (RNAi). Immunohistochemistry (IHC) staining of 127 cases of MEC tissues was scored with the expression index (EI). The EI of MDR1 and MRP1 (or nuclear MRP1) was analyzed with Spearman's rank correlation analysis. Using multiple tumor tissue assays, the location of MRP1 in other tissues was checked by HIC. Luciferase reporter assays of MDR1 promoter was carried out to check the connection between MRP1 and MDR1 promoter.

**Results:**

MRP1 downregulation led to a decreased MDR1 expression in MC3/5FU cells which was caused by decreased activity of MDR1 promoter. IHC study of 127 cases of MEC tissues demonstrated a strong positive correlation between nuclear MRP1 expression and MDR1 expression. Furthermore, IHC study of multiple tumor tissue array sections showed that although nuclear MRP1 widely existed in MEC tissues, it was not found in normal tissues or other tumor tissues.

**Conclusions:**

Our findings indicate that nuclear MRP1 contributes to MDR mainly through regulating MDR1 expression in MEC. And the unique location of MRP1 made it an available target in identifying MEC from other tumors.

## Introduction

Mucoepidermoid carcinoma (MEC) is the most common primary malignant salivary gland tumor in occidental and Chinese population [1], [2], [3]. It comprises about 10% of all salivary gland neoplasms and about 35% of malignant lesions [4]. In pediatric patients, MEC comprises about 16% of all salivary gland neoplasms and about 51% of malignant lesions [5]. MEC is divided into low, intermediate and high grade on the basis of morphological and cytological features. Low and intermediate grade MEC usually have a high survival rate, while high grade MEC usually has very poor prognosis, the 5-year survival rate of the high grade MEC victims is only about 30% [6], [7]. MEC usually has low chemosensitivity, so chemotherapy is mainly used for unresectable or metastatic cases [8].

Multidrug resistance protein 1 (MDR1/P-glycoprotein/ABCB1) and multidrug resistance-related protein 1 (MRP1/ABCC1), both belonging to the ATP-binding cassette superfamily of membrane-bound transporters, are two genes that are found to be highly related to multidrug-resistance of cancer cells. The human MDR1 spans over 100 kb on human chromosome 7q21 and the human MRP1 spans over 194 kb on chromosome 16p13.1. The amino-acid sequence of the two genes resembles only to a modest extent (∼15%). The substrates for MDR1 are commonly hydrophobic drugs having neutral or positive charge and MRP1 commonly transports neutral and anionic hydrophobic natural products. In contrast to MDR1, MRP1 also transports glutathione (GSH),drugs conjugated to glutathione,glucuronate, or sulfate [9], [10]. Due to its extraordinary transport properties, MRP1/ABCC1 contributes to several physiological functions and pathophysiological incidents, such as inflammatory responses and oxidative stress [10]. MRP1 was firstly discovered in a multidrug-resistant small-cell lung cancer cell line [11], afterward the increased expression of MRP1 has been reported in a variety of hematological and solid tumors. The location of MRP1, which is predominantly on the cytomembrane of tumor cells, also suggests its transporting role in clinical drug-resistance. Despite the comprehensive study of MRP1, the crystal structure and the transport mechanism of MRP1 remains elusive, and the broadly existed splice variants and mutations of MRP1 in tumors also make its function unpredictable [10], [12].

In our previous study, we found nuclear MRP1 in MEC cells for the first time and proved that nuclear translocation of MRP1 was one of the reasons leading to multidrug-resistance of MC3/5FU cells [13]. To explore why the downregulation of MRP1 reversed the resistance to taxol which is not the substrate of MRP1 [13] but the substrate of MDR1 [9], we investigated MDR1 expression in MC3/5FU cells when MRP1 was downregulated through short-hairpin RNAs (shRNA) in this study. We also investigated the expression pattern of MDR1 and MRP1 in 127 cases of MEC patients by immunohistochemistry and explored the correlation between MRP1 and MDR1 in MEC. Our results implied that MRP1 may not affect chemosensitivity of MEC though transporting drugs directly, but though modulating the expression of MDR1.

## Methods

### Patient materials

Human MEC tissues were obtained from 127 patients who had received no pretreatment before surgery at the Department of Oral and Maxillofacial Surgery, School of Stomatology, the Fourth Military Medical University, from July 2006 to July 2011. After being formalin-fixed, paraffin-embedded, the MEC specimens were diagnosed and identified by the Department of Oral Histology and Pathology, School of Stomatology, the Fourth Military Medical University. The protocol and performance of our research was approved and monitored by the Ethics Committee of the Fourth Military Medical University. All patients involved have been informed and signed the consent to participate this research.

### Immunohistochemistry (IHC)

The multiple tumor tissue array section (AM®00C13) had 96 array cores in total which contained 48 different tumors and their corresponding normal tissues (AoMei Biotechnology Co., Xi'an, China,). The section thickness was 5 µm and the array core diameter was 1 mm. MEC tissue sections (5 µm) of the 127 patients and the multiple tumor tissue array sections were deparaffinized in xylene and rehydrated. Sections for MRP1 staining were pretreated by boiling in Tris/EDTA (1 mmol/l in distilled water, pH 9.0, Zhongshan, Beijing, China) for 20 minutes, sections for MDR1 staining were pretreated by boiling in citrate buffer (0.01 mol/l in distilled water, pH 6.0, Zhongshan, Beijing, China) for 2 min in a steaming pressure cooker. Endogenous peroxidase activity was then blocked by 0.3% (v/v) H_2_0_2_ in methanol for 20 minutes. Immunohistochemistry assay (IHC) was performed with Streptavidin Biotin Peroxidase Detection kit (Zhongshan, Beijing, China) according to the manufacturer's protocol. Mouse anti-MRP1 and mouse anti-MDR1 monoclonal antibodies, as the primary antibody, were obtained from Maixin-Bio, Fuzhou, China (1∶40). The slides were stained with 3,3′-diaminobenzidine (DAB) and counterstained with haematoxylin, then observed under a microscope (Leica DMI6000 B Fully Automated Inverted Research Microscope, Germany) with 200×magnification.

### Assessment of immunostaining

Staining was evaluated independently by two of the authors (BL Cai, Y Liu). Results were scored in a qualitative and a quantitative way. The qualitative score (QLS) was the average of each cell's score (or each cell nucleus's score) which was evaluated relative to the salivary acini and graded as follows: 0 = below the normal epithelium or negative; 1 = resembling the normal epithelium with weak staining; 2 = distinctly enhanced staining; 3 = strong staining. Cells (or cell nuclei) stained stronger than the salivary acini were evaluated as positive. The quantitative score (QNS) was determined by the percentage of positive cells in all the cells. The nuclear quantitative score (NQNS) was determined by the percentage of positive cell nuclei in all positive cells. At least 10^3^ cells in three different parts of one sample were analyzed and scored: 0 = 0–5% positive; 1 = 6–25% positive; 2 = 26–50% positive; 3 = 51–100% positive. For statistical analysis, the expression index (EI) for each sample was calculated from both scores by the equation: QLS·QNS (or QLS·NQNS) and thus gave values from 0 to 9.

### lmmunofluorescent histochemical staining

The pretreatment of the sections in immunofluorescent histochemical staining was the same as in IHC. In immunofluoscent histochemical staining, fluorescence second antibody was used (Fluorescein-Conjugated AffiniPure Goat Anti-Mouse IgG, Zhongshan, Beijing, China) (15 µg/ml, 37°C, 30 min). The nuclei were counterstained with 4′, 6-diamidino-2-phenylindole (1 µg/ml, room temperature, 2 min). Samples were examined under a FluoView™ FV1000 Confocal Microscope (Olympus Corporation, Japan) with a ×40 (numerical aperture, 1.2) oil immersion lens. Images were processed with FV10-ASW software (Olympus).

### Cell culture

High metastatic human mucoepidermoid carcinoma cell line MC3 [13], 5FU-resistant subline MC3/5FU [13], the transfected stable clones MC3/5FU–S [13] cells (transfected with specific shRNA for MRP1), MC3/5FU-NS cells (transfected with non-specific shRNA) [13] and MEC-1 [14] were all established and stored in our laboratory. All the cells were cultured as in previous description.

### Construction of short hairpin RNA (shRNA) expressing plasmid and stable gene transfection

The MC3/5FU cells were transfected with specific shRNA for MRP1 and non-specific shRNA, the sequences of the shRNA and the method of selecting the stable clones were the same as in previous description [13].

### Reverse transcription-polymerase chain reaction (RT-PCR)

According to the manufacturer's protocol, total RNA was isolated from cells respectively by using Total RNA extraction kit I (Omega, USA). Total RNA was reversely transcribed with opposite transcriptase (Takara, Japan). The first-strand cDNA was used as the template for quantitative real-time RT-PCR or semi-quantitative RT-PCR analysis. GAPDH was used as an internal control to normalize variances. The sequences of the primers and length of PCR products were shown in Table 1. Quantitative RT-PCR experiments were performed by using SYBR® Premix Ex TagTMII (Takara, Japan) under the following conditions: 30 s hot start at 95°C; continuation with 45 cycles of 5 s at 95°C, 34 s at 56°C, and 15 s at 72°C. The relative expression levels were calculated by using the comparative threshold cycle (ΔΔCT) method. To ensure specificity of the PCR product amplification, the melting curves were analyzed. Semi-quantitative RT-PCR experiments were performed by using the Premix Taq® Version 2.0 (Takara, Japan). The cycling program for the 25 µl reaction mixture was performed as follows: 94°C for 5 minutes as hot start, 94°C for 30 s, 56°C for 40 s, 72°C for 45 s, and 72°C for 10 minutes. The PCR products were analyzed by electrophoresis in a 1.5% TAE-agarose gel with ethidium bromide (0.5 mg/ml TE buffer) and photographed using Bio-Rad Gel Doc™ XR system (Bio-Rad, Hercules, CA).

**Table 1:** Primer sequences and PCR conditions.

Gene	Oligonucleotide sequence (5′-3′)	bp	Cycles
MRP1(ABCC1)	F: CATTCAGCTCGTCTTGTCCTG	72	34
	R:GGATTAGGGTCGTGGATGGTT		
MDR1(ABCB1)	F: AGACATGACCAGGTATGCCTAT	158	34
	R: AGCCTATCTCCTGTCGCATTA		
GAPDH	F:CAACTACATGGTTTACATGTTC	181	30
	R:GCCAGTGGACTCCACGAC		

MRP1, nuclear multidrug-resistance related protein 1; MDR1, Multidrug-resistance protein 1.

### Western blot analysis

The protein samples were separated and analyzed as in the previous description [13].

### Immunofluorescent Confocal Laser-Microscopy

MRP1 was localized in the cells as previously described [13].

### Transient Transfections and luciferase reporter assays

P-MDR1, containing 241 bp *MDR1* promoter (spanning the −198 to +43 region), was PCR-generated and subcloned into the pGL3 luciferase reporter construct (Promega, Madison, WI). To avoid the affection of GFP, two RNA interference (RNAi) candidate target sequences of human MRP1 and one negative control were designed and synthesized, then inserted into the pGPU6/Neo plasmid (Shanghai Gene Pharma Co. Ltd Shanghai, P.R.China). The first specific shRNA sequence: 5′-GGTCACCCATGAGCTTCTTTGttcaagagaCAAAGAAGCTCATGGGTGACCtt-3′ (pGPU6/Neo-shRNA-MRP1, the transiently transfected cells are named MC3/5FU-S), The second specific shRNA sequence: 5′-GGAAGAAGGAATGCGCCAAGATtcaagagATCTTGGCGCATTCCTTCTTCCtt-3′ (pGPU6/Neo-shRNA-MRP1-1, the transiently transfected cells are named MC3/5FU-S1), The non-specific shRNA sequence: 5′-gTTCTCCGAACGTGTCACGTcaagagattACGTGACACGTTCGGAGAAtt-3′ was used as a negative control (GPU6/Neo-shRNA-NS, the transiently transfected cells are named MC3/5FU-NS). All constructs were cotransfected into MC3/5FU cells with luciferase reporter constructs of P-MDR1 (or with empty vector pGL3-basic as a control) for 48 hours. Transfection efficiency was normalized by cotransfection with pRL-CMV, expressing Renillar luciferase (Promega). Transfection was done directly in 6-well cell culture plates with 10^6^ cells/well plated in growth medium 1 day before, using Lipofectamine 2000 (Invitrogen, Carlsbad, California, USA). Each well contained plasmids of 2 µg P-MDR1-luc or pGL-3, 2 µg of pGPU6/Neo-shRNA-MRP1, pGPU6/Neo-shRNA-MRP1-1 or pGPU6/Neo-shRNA-NS, and 0.03 µg of pRL-CMV. The luciferase activities were then measured by Dual-Glo System (Promega) on a TopCount (Packard Instrument, Meriden, CT). The results from three independent experiments, with each in triplicate, were expressed as fold induction after normalization with transfection efficiency and pGL3-Basic vector control.

### Statistical analysis

All data were expressed as means ± standard error (SEM). Student's t-test and one-way ANOVA (LSD) were used for determining the significance of difference in comparisons. The relationship between the EI of MDR1 and MRP1 (or nuclear MRP1) was analyzed with Spearman's rank correlation analysis and the scatter diagram was made to check whether linear trend existed between MDR1 and MRP1 expression. The correlation coefficient (r) was calculated to measure the correlation degree between MDR1 and MRP1. Calculations were carried out by software SPSS version 12.0, P<0.05 was considered statistically significant.

## Results

### Down-regulation of MRP1 decreased MDR1 expression in MC3/5FU cells by decreasing the Transcriptional Activity of the MDR1 Promoter in vitro

**Figure 1:**
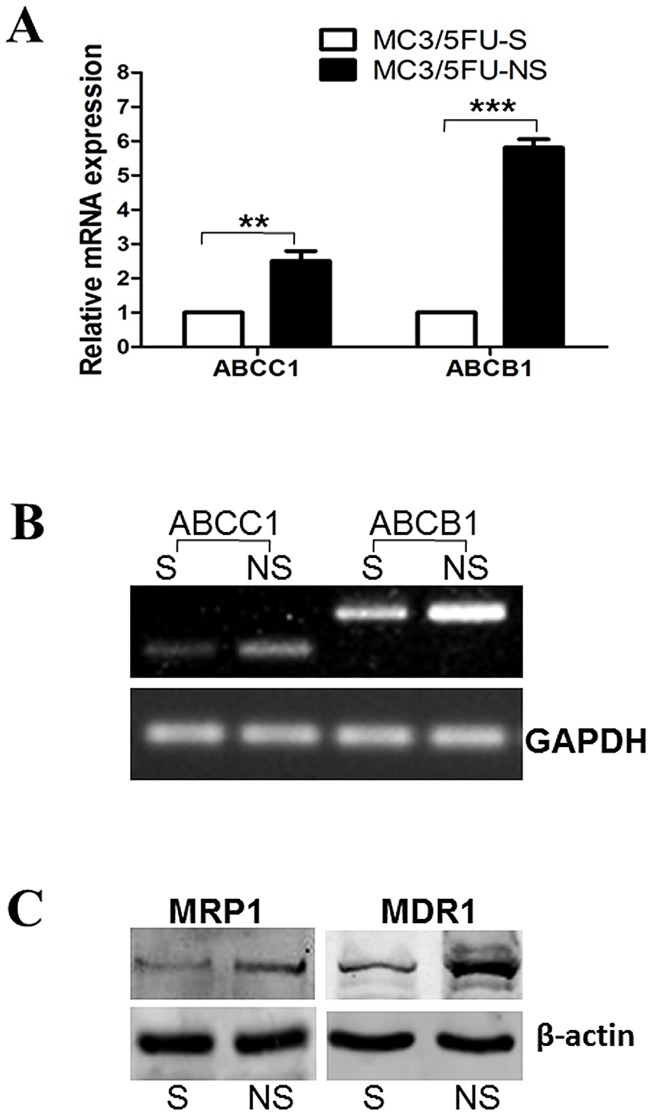
Downregulation of MRP1 decreased MDR1 expression in MC3/5FU cells. MC3/5FU cells were transfected with plasmids containing a ABCC1/MRP1 specific shRNA and a non-specific control shRNA, the resulting clones were MC3/5FU–S (S for silenced) and MC3/5FU-NS (NS for non-silenced). Total RNA and total protein were extracted from the 2 cell lines and then subjected to RT-PCR and Western blot respectively. (A) Quantitative real-time RT–PCR result (n = 3). The values were nomalized with GAPDH. When ABCC1/MRP1 mRNA was significantly down-regulated in MC3/5FU-S cell, ABCB1/MDR1 mRNA was simultaneously significantly down-regulated. (B) Representative RT-PCR results (n = 3). GAPDH was used as loading control. (C) Representative Western-blot results. β-actin was used as loading control. ** P<0.01 vs MC3/5FU-S, *** P<0.001 vs MC3/5FU-S.

To further explore the function of MRP1 in MEC, we decreased the MRP1 expression in MC3/5FU cells by RNA interference (RNAi). In our previous study, MRP1 was found mainly in the nuclei of MC3/5FU cells and the decreased MRP1 was mainly the nuclear MRP1. We thought that the decreased multidrug-resistance of MC3/5FU cells was mainly caused by the decreased nuclear MRP1 expression. Compared with the non-specific shRNA transfected MC3/5FU-NS cells, MRP1 mRNA expression in MC3/5FU-S cells was significantly reduced by 65±5% (N = 3, P<0.01). Interestingly, the mRNA expression of MDR1 was reduced by 83±0.6% (N = 3, P<0.01) at the same time (Figure 1A). RT–PCR and Western blot confirmed the results (Figure 1B and 1C).

**Figure 2:**
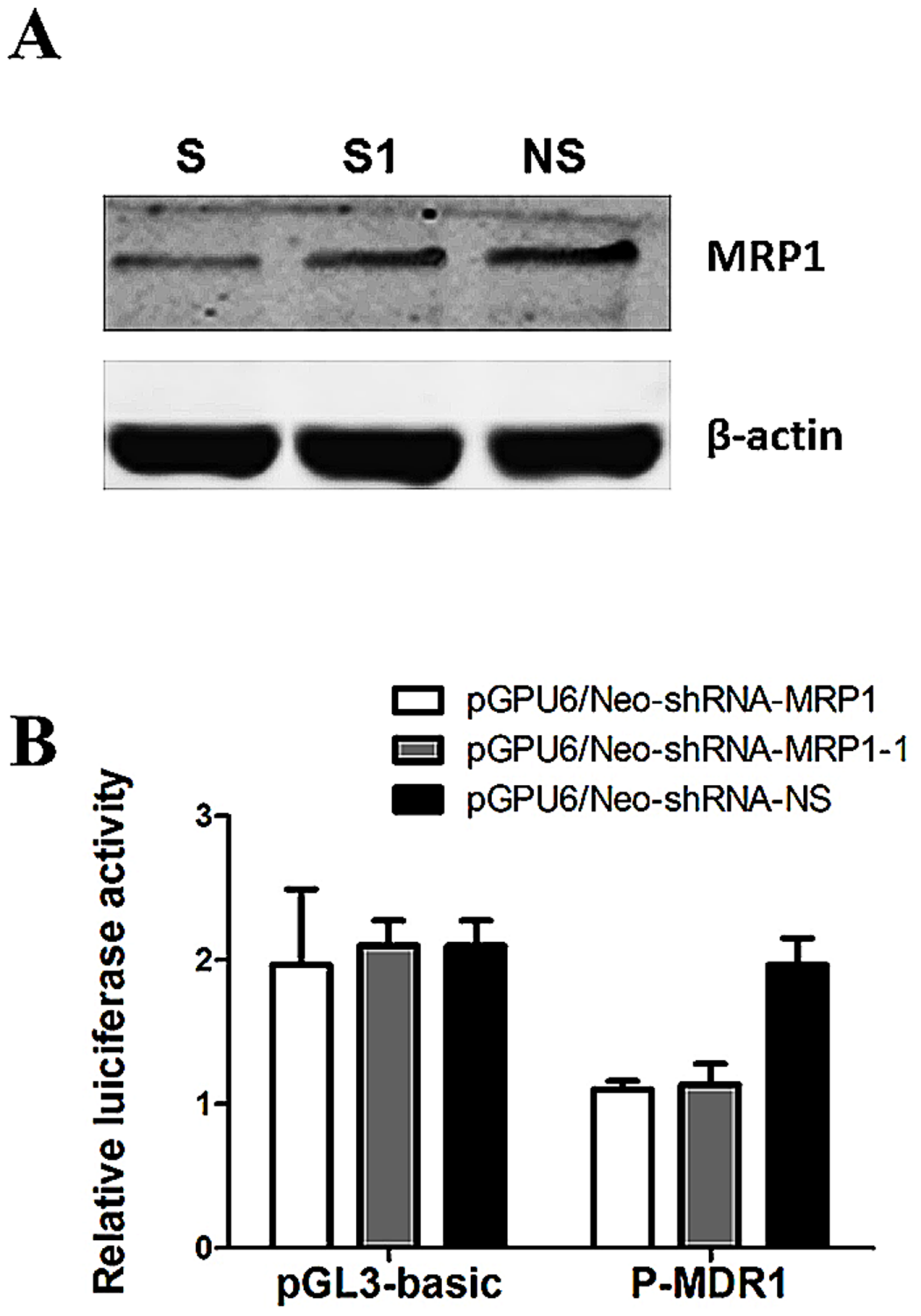
Down-regulation of MRP1 decreased the Transcriptional Activity of the MDR1 Promoter in vitro. (A) MC3/5FU cells which were transiently transfected with pGPU6/Neo-shRNA-MRP1 were named MC3/5FU-S (S), MC3/5FU cells which were transiently transfected with pGPU6/Neo-shRNA-MRP1-1 (S1) were named MC3/5FU-S1 (S1), MC3/5FU cells which were transiently transfected with pGPU6/Neo-shRNA-NS were named MC3/5FU-NS (NS). After MC3/5FU cells were transiently transfected with shRNA for 48 hours, MRP1 expression of MC3/5FU-S and MC3/5FU-S1 cells was decreased compared to MC3/5FU-NS cells. (B) All shRNAs were cotransfected into MC3/5FU cells with luciferase reporter constructs of P-MDR1 (or with empty vector pGL3-basic as a control) for 48 hours. Transfection efficiency was normalized by cotransfection with pRL-CMV, expressing Renillar luciferase. Compared with the activity from the cotransfection with P-MDR1 and pGPU6/Neo-shRNA-NS, the transcriptional activity of the reporter gene was significantly decreased by cotransfection with P-MDR1 and pGPU6/Neo-shRNA-MRP1 (n = 3). And compared with the activity from the cotransfection with P-MDR1 and pGPU6/Neo-shRNA-NS, the transcriptional activity of the reporter gene was significantly decreased by cotransfection with P-MDR1 and pGPU6/Neo-shRNA-MRP1-1 (n = 3). The promoter-less pGL3-Basic that was cotransfected with pGPU6/Neo-shRNA-MRP1, pGPU6/Neo-shRNA-MRP1-1 or pGPU6/Neo-shRNA-NS showed a background activity and was not significantly changed by the sh-RNA expression vector.

We next performed a functional study in MC3/5FU cells to determine the effect of MRP1 on the ABCB1 gene. After MC3/5FU cells were transiently transfected with shRNA for 48 hours, MRP1 expression of MC3/5FU-S and MC3/5FU-S1 cells was decreased compared to MC3/5FU-NS cells (Figure 2A). All shRNAs were cotransfected into MC3/5FU cells with luciferase reporter constructs of P-MDR1 (or with empty vector pGL3-basic as a control) for 48 hours. Transfection efficiency was normalized by cotransfection with pRL-CMV, expressing Renillar luciferase. Luciferase activities were measured with a luminometer. Compared with the activity from the cotransfection with P-MDR1 and pGPU6/Neo-shRNA-NS, a 44.1% decrease in the transcriptional activity of the reporter gene was obtained by cotransfection with P-MDR1 and pGPU6/Neo-shRNA-MRP1, (P = 0.01, figure 2B). A 42.4% decrease in the transcriptional activity of the reporter gene was obtained by cotransfection with P-MDR1 and pGPU6/Neo-shRNA-MRP1-1 compared with the activity from the cotransfection with P-MDR1 and pGPU6/Neo-shRNA-NS (P = 0.02, figure 2B). The promoter-less pGL3-Basic that was cotransfected with pGPU6/Neo-shRNA-MRP1, pGPU6/Neo-shRNA-MRP1-1 or pGPU6/Neo-shRNA-NS showed a background activity and was not changed by the sh-RNA expression vector.

### MRP1 was localized in the cell nuclei of MEC tissues

**Figure 3:**
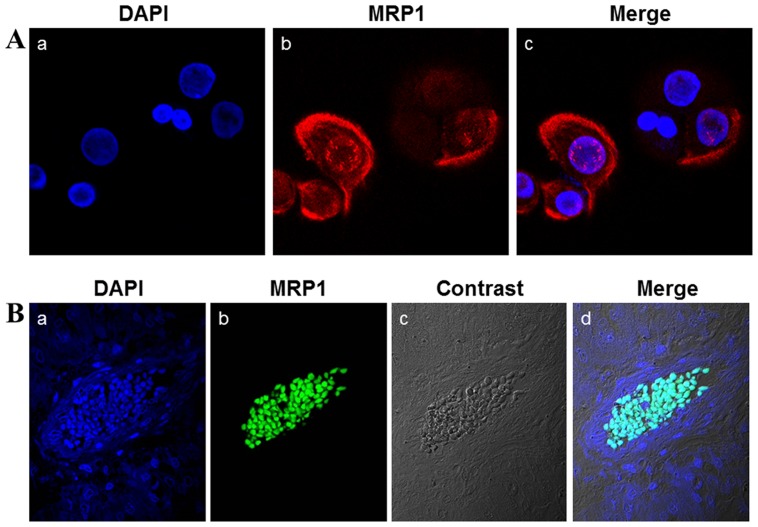
Nuclear MRP1 was a widespread phenomenon in MEC tissues. To exclude the possibility that MRP1 was expressed on the surface of nuclear membrane of MEC cells, the location of MRP1 was layer scanned by immunofluorescent confocal laser-microscopy. (A) 5×10^4^ MEC-1 cells were cultured on glass coverslips for 24 hours and then subjected to immunofluorescent cytochemistry staining. The cells were reacted with mouse monoclonal antibody specific for human MRP1, and were then stained with DyLight™594-conjugated AffinPure Dongkey Anti-Mouse IgG (b) and counterstained with DAPI (a) to reveal nuclei. (c) Merged pictures. The merged pictures showed that MRP1 was localized on the cytomembrane and the nuclei of MEC-1 cells which were derived from MEC tissue whose MRP1 expression was negligible in the immunohistochemistry (IHC) study. (B) MEC tissue with obvious nuclear MRP1 expression by IHC was subjected to immunofluorescent histochemical staining. MRP1 were reacted with mouse monoclonal antibody specific for human MRP1, then stained with Fluorescein-Conjugated AffiniPure Goat Anti-Mouse IgG second antibody (b) and counterstained with DAPI (a) to reveal nuclei, (c) phase contrast picture of MEC tissue, (d) merged picture. The merged picture showed that MRP1 was mainly localized in the nuclei of cells of MEC tissues.

To confirm MRP1 was expressed in the nuclei of MEC cells and to exclude the possibility that the nuclear staining of MRP1 was caused by the nonspecific bonding of the antibody, we used primary and second antibodies from different companies (compared with reference 13) in the immunofluorescent histochemical staining and IHC staining. In immunofluorescent histochemical study which was layer scanned by using of the laser scanning confocal microscope, the cyan section of the merged picture showed that MRP1 was strongly expressed in the nuclei of MEC cells (Figure 3B).

### The expression of MDR1, especially the nuclear MRP1, was highly correlated with the expression of MRP1 in MEC tissues

**Figure 4:**
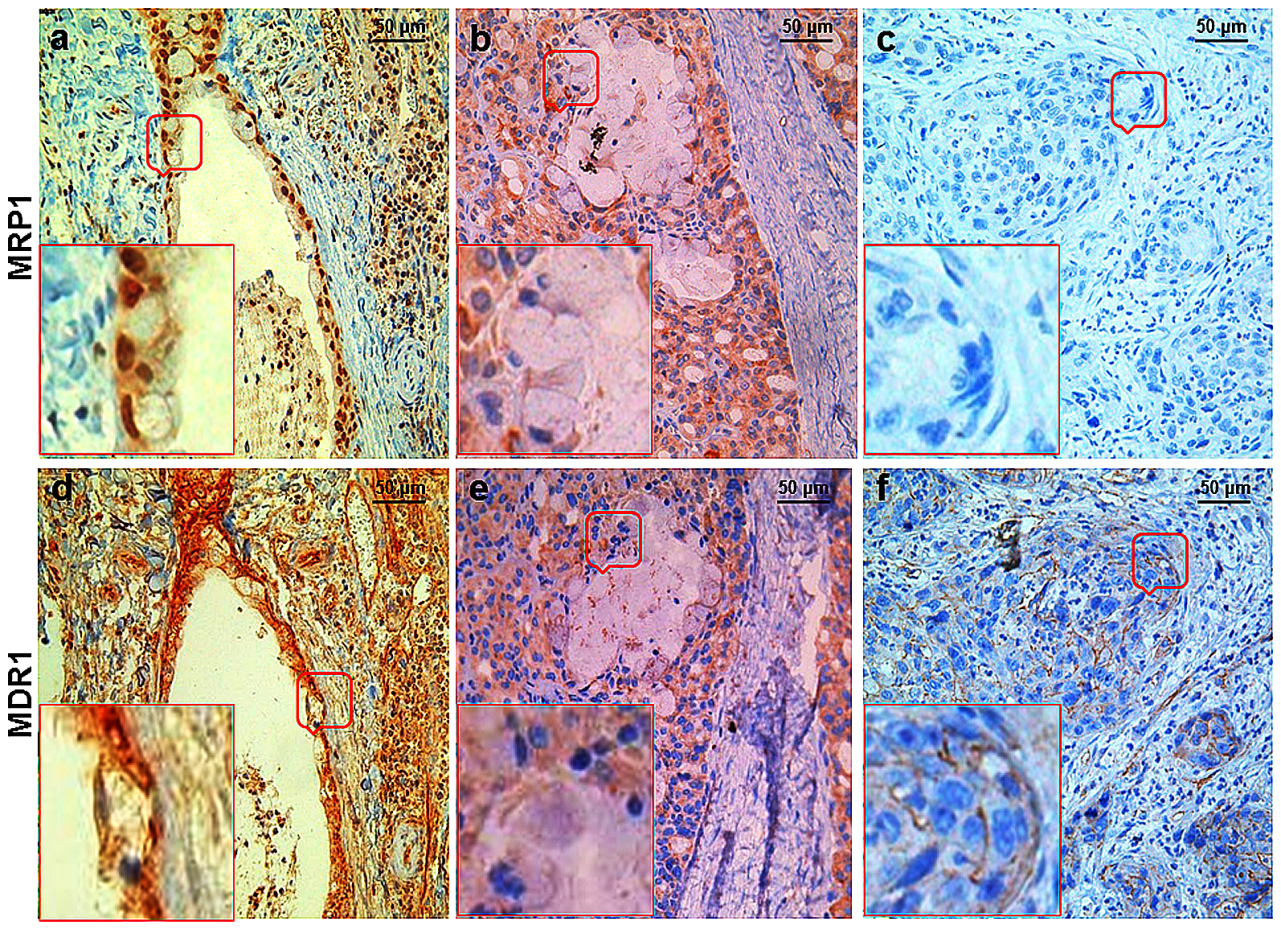
Strong positive correlation was found between MDR1 and MRP1/nuclear MRP1 expression in MEC tissues. Immunohistochemistry (IHC) staining was conducted to determine the localization and expression of MRP1 in formalin-fixed, paraffin-embedded mucoepidermoid carcinoma tissues using anti-MRP1 antibody. Results were scored in qualitative and quantitative way. The tissues were counterstained with hematoxylin. Brownish staining indicated MRP1 (a, b, c) or MDR1 (d, e, f) expression in the tissues. In the strongly MRP1/nuclear MRP1 stained MEC tissues (a), MDR1 was also strongly expressed (d). And MDR1 was higher expressed in cells with higher nuclear MRP1 expression compared with cells with lower nuclear MRP1 expression. In the moderately MRP1 expressed MEC tissues (b), moderately expressed MDR1 (e) were found both in the cytoplasm and cytomembrane of MEC cells. In the weakly MRP1 expressed MEC tissues (c), faint expression of MDR1 (f) was detected on the cytomembrane of MEC cells (200×magnification).

Expression levels and patterns of MRP1 and MDR1 were analyzed in 127 MEC patients without pretreatment before surgery. In the immunohistochemistry study, MRP1 could be found in the cytomembrane, cytoplasm and nuclei. Nuclear MRP1 was observed in 67% specimens (85 patients). MDR1 was found in the cytomembrane or cytoplasm in MEC cells. Interestingly, MDR1 was always more expressed in the cells where MRP1 was more expressed. When MRP1 was mainly expressed in the nuclei, MDR1 was highly expressed and mainly localized on the cytomembrane (Figure 4a, d) which indicated higher transport ability of these cells. When MRP1 was mainly localized in the cytoplasm, MDR1 was less expressed and mainly localized in the cytoplasm (Figure 4b, e). In specimens whose MRP1 expression was negligible, MDR1 was only weakly expressed on the cytomembrane (Figure 4c, f).

The primary cells (MEC-1) were derived from the MEC tissue whose MRP1 expression was negligible in IHC study (Figure 4c). To further explore the MRP1 expression pattern in MEC, we localized MRP1 in MEC-1 cells by immunofluorescent cytochemistry. Our results showed MRP1 expressed with different intensities in different MEC cells, which accorded with the characteristics of primary culture cells. Yet, MRP1 could still be found in the nuclei of some MEC cells (Figure 3A). Our results implied that nuclear MRP1 might be a widely existing phenomenon in MEC.

**Figure 5:**
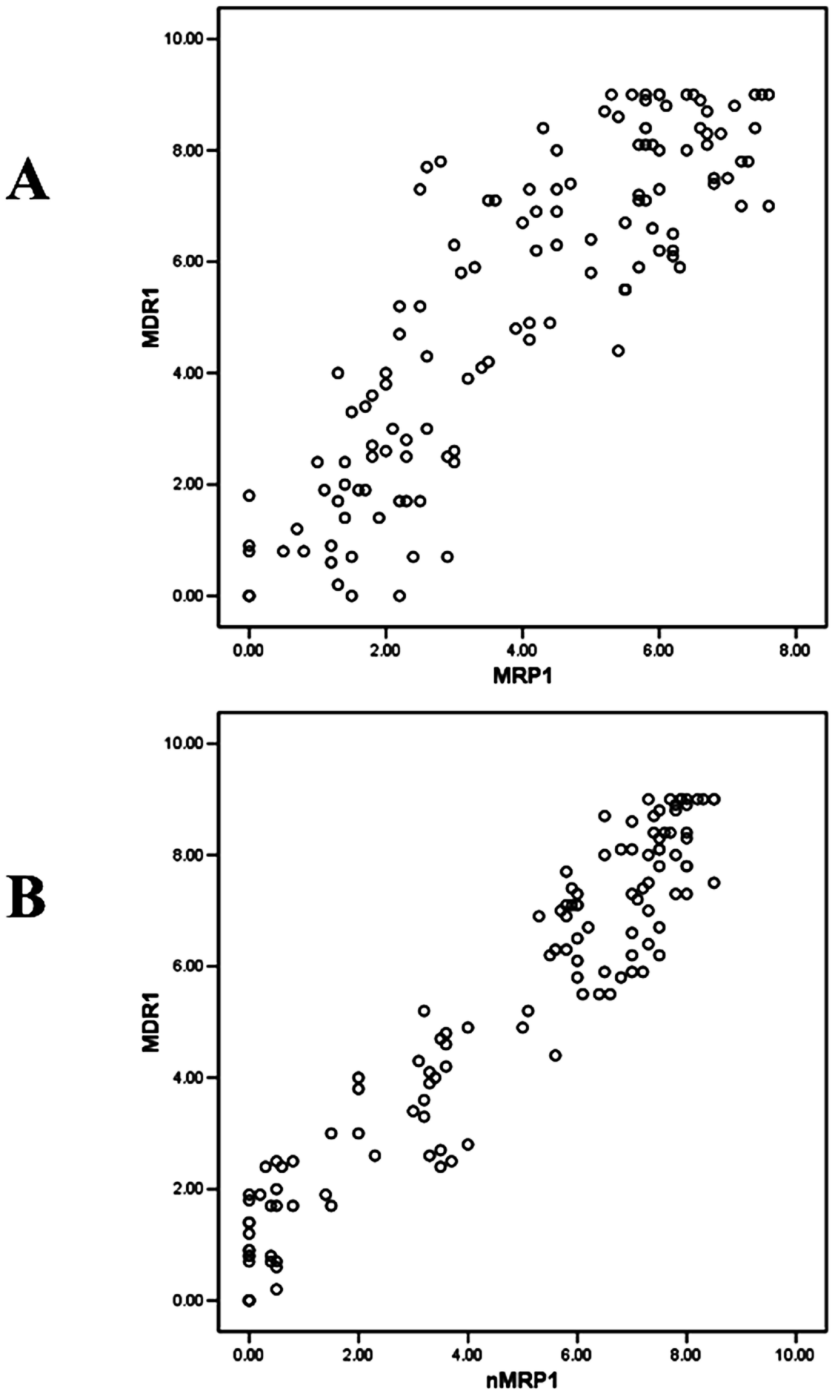
Obvious linear correlation was found between MDR1 and MRP1/nuclear MRP1 expression in MEC. The scatter diagram was made by SPSS, the X-axis represented the expression of MRP1 or nuclear MRP1 (nMRP1) and the Y-axis represented the expression of MDR1. (A) There was obvious linear trend between MDR1 and MRP1 expression in MEC tissues. (B) The linear trend between MDR1 and nuclear MRP1 expression in MEC tissues was more obvious.

The relationship between the EI of MDR1 and MRP1/nuclear MRP1 in MEC tissues was analyzed with Spearman's rank correlation analysis. An obvious linear trend between MDR1 and MRP1 (or nuclear MRP1) expression in MEC was found (Figure 5). There was a positive correlation between MDR1 and MRP1 expression in MEC tissues (r = 0.848, p<0.01). Furthermore, a stronger positive correlation was found between MDR1 and nuclear MRP1 in MEC tissues (r = 0.931, p<0.01).

### No nuclear MRP1 expression was found in the normal tissues and tumor tissues in the multiple tumor tissue arrays

IHC staining was conducted to determine the localization and expression of MRP1 in the tumors and their corresponding normal tissues of the multiple tumor tissue assays. Each picture corresponded with one kind of tissue. The numbers of the pictures of Figure S1 corresponded to the numbers in the Table S1. In all these tissues of the multiple tumor tissue arrays, MRP1 was only found on the cytomembrane or in the cytoplasm, no MRP1 was found in the cell nuclei. The additional files showed this in more details.

## Discussion

In our previous study, we found that MRP1 was mainly located in the nuclei of MC3/5FU cells [13]. In addition, MRP1 downregulation reversed the resistance of MC3/5FU cells to taxol [13] which was not the substrate of MRP1 but the substrate of MDR1 [9]. In this study, we explored the relationship between MRP1 and MDR1 in MEC and partly explained how MRP1 leaded to MDR in MEC.

In the IHC study, nuclear MRP1 was found in 67% specimens, which demonstrated that nuclear MRP1 was a widespread phenomenon in MEC. Our results re-confirmed the existence of nuclear MRP1 and also implied that nuclear MRP1 was not a unique phenomenon in the multidrug-resistant cell line (MC3/5FU). Moreover, when we used the immunofluorescent cytochemistry which was a more sensitive and specific method to localize protein, MRP1 was also localized in the nuclei (Figure 3A) of a part of the primarily cultured cell line MEC-1, whose corresponding MEC tissue showed a negligible MRP1 expression (Figure 4C). This result further suggested that nuclear MRP1 might exist in most of MEC tissues. To clarify whether nuclear MRP1 exist in other kinds of tumors, we examined multiple arrays of tumor tissues including 48 different tumor tissues and their corresponding normal tissues and did not find any nuclear MRP1 expression in these tissues (Figure S1). Whether nuclear MRP1 exists in other kinds of tumors still needs further study, but our results showed that nuclear MRP1 was a widespread phenomenon in MEC and its unique location made it an available target for distinguishing MEC from other tumors.

The human MRP1 gene is composed of 31 exons. Polymorphisms of MRP1 which included splice variants or nucleotide mutation were found not only in tumor cells [12], [15] but also in normal cells [12], [16], [17]. Different mutations lead tovarious kinds of variants, showing distinguished functions [17], [18], [19]. Even the same MRP1 mutation in different cells has different functions [16]. Although the protein expression plays an important role in chemotherapy, it is that the genotype of the MRP1, rather than the protein expression of MRP1, determines the response to various types of chemotherapy [17]. To date, at least 15 naturally occurring mutations and more than 2350 single nucleotide polymorphisms have been identified in MRP1. However, most of these nucleotide polymorphisms' function is unknown [10]. The unique existence of nuclear MRP1 could highly improve the prognosis accuracy of MEC and may suggest its unique sequence even structure which could be used to design targeted therapy against MEC. If the unique sequence of MRP1 in MEC was found, we could design specific shRNA, using synthetic materials or natural carriers (viruses and bacteria) to delivery, to exclusively decrease the expression of MRP1 in MEC cells without affecting normal cells [20], [21], [22]. The chemotherapy efficiency could be increased after the decrease of MRP1 in MEC cells [13]. If the unique structure of MRP1 in MEC was found, we could design monoclonal antibody, targeting the unique structure of MRP1, to block MRP1 of MEC cells. For example, trastuzumab, a monoclonal antibody against HER2 protein, results in improved DFS and OS in breast cancers patients in both the adjuvant and metastatic settings [23], [24], [25].

In the immunochemistry study, we found that MDR1 was always expressed higher in the cells where MRP1 was obviously stained. Moreover, when MRP1 was mainly expressed in the nuclei, MDR1 was more expressed and mainly localized on the cytomembrane (Figure 4) which meant higher drug transporting ability of these cells. The Spearman's rank correlation analysis also showed that, compared with the positive correlation between MDR1 and MRP1 expression, a stronger positive correlation was found between MDR1 and nuclear MRP1 (Figure 5). Our RNAi experiment further demonstrated that the change of MDR1 was due to the change of nuclear MRP1 which acted as a functional protein itself, rather than the markers of the functional protein. Luciferase reporter assays indicated that downregulation of MRP1 decreased the activity of MDR1 promoter in multidrug-resistant MC3/5FU cells. But how does MRP1 affect the expression of MDR1 still needs our further study. Considering the nuclear localization and functional role of MRP1 in regulating MDR1, a more plausible hypothesis to explain the positive relationship between MRP1 and MDR1 is that MRP1, as a glutathione (GSH) transporter, participated in the mitogen-activated protein kinase (MAPK) pathway through affecting the local GSH concentration in the cells. The MAPK family is serine/threonine kinases which mediate intracellular signal transduction pathways. This family contains three sequence and structurally similar classes of enzymes: the extracellular signal regulated kinases (ERKs), Jun N-terminal kinases (JNKs) and P38 kinases [26]. P38, which activates with transcription factors, protein kinases, and nuclear proteins, could mediate diverse responses such as cell differentiation, senescence, cell-cycle arrest, apoptosis, cytokine production, or regulation of RNA splicing [27]. Inactivating p38 could directly or indirectly act on ATP-binding pocket [28] to enhance the chemosensitivity of some tumors in vivo and in vitro [29], [30]. Several evidences proved that p38 inhibition could enhance the treatment effect in acute promyelocytic leukemia cells [31] chronic myeloid leukemia cells [32] and multiple myeloma cells [33]. In MEC, MRP1 overexpression causes the depletion of intracellular GSH which causes the downregulation of MKP-1 [34]. MKP-1, as a nuclear enzyme, is able to dephosphorylate and inactivate the stress-activated p38-MAPK [35]. MKP-1 downregulation could enhance the phosphorylation of p38-MAPK which leads to the upregulation of MDR1 expression by increasing activator protein-1 (AP-1) activity then increases the MDR of tumor cells [36]. We thought the nuclear translocation of MRP1 changed local GSH concentration, which affected MKP-1 expression, more efficiently in the nuclei. This explained why down-regulation of MRP1 in MC3/5FU increased the drug sensitivity without quantitative alteration and why the relationship between nuclear MRP1 and MDR1 was stronger than the relationship between total MRP1 and MDR1.

## Conclusions

In conclusion, our study found that MRP1 led to multidrug-resistance of MEC mainly via regulating MDR1 expression. And the unique location of MRP1 made it an available target in identifying MEC from other tumors.

## Supporting Information

Figure S1 **No nuclear MRP1 expression was found in the tissues of the multiple tumor tissue assays in the HIC staining.** IHC staining was conducted to determine the localization and expression of MRP1 in the tumors and their corresponding normal tissues of the multiple tumor tissue assays. Each picture corresponded with one kind of tissue. The numbers in the pictures corresponded to the numbers in the table of Additional file 1. No nuclear MRP1 expression was found in these tissues of the multiple tumor tissue assays in the HIC staining.
(PDF)

Materials and Methods S1 **Immunohistochemistry of the multiple tumor tissue arrays.**
(DOCX)

Table S1 **Detailed information of tissue array panel.**
(DOCX)
