# Supplementary material for: Nuclear Multidrug-Resistance Related Protein 1 Contributes to Multidrug-Resistance of Mucoepidermoid Carcinoma Mainly via Regulating Multidrug-Resistance Protein 1: A Human Mucoepidermoid Carcinoma Cells Model and Spearman's Rank Correlation Analysis
Source: PLoS One. 2013 Aug 27;8(8):e69611. doi: 10.1371/journal.pone.0069611 (PMC3754958; doi:10.1371/journal.pone.0069611)
Supplement: Figure S1 — No nuclear MRP1 expression was found in the tissues of the multiple tumor tissue assays in the HIC staining. IHC staining was conducted to determine the localization and expression of MRP1 in the tumors and their corresponding normal tissues of the multiple tumor tissue assays. Each picture corresponded with one kind of tissue. The numbers in the pictures corresponded to the numbers in the table of Additional file 1. No nuclear MRP1 expression was found in these tissues of the multiple tumor tissue assays in the HIC staining. (PDF) [file pone.0069611.s001.pdf]

Supporting information 3

Figure S1: No nuclear MRP1 expression was found in the tissues of the multiple tumor tissue assays in the HIC staining

IHC staining was conducted to determine the localization and expression of MRP1 in the tumors and their corresponding normal tissues of the multiple tumor tissue assays. Each picture corresponded with one kind of tissue. The numbers in the pictures corresponded to the numbers in the Table S1. No nuclear MRP1 expression was found in these tissues in the HIC staining.

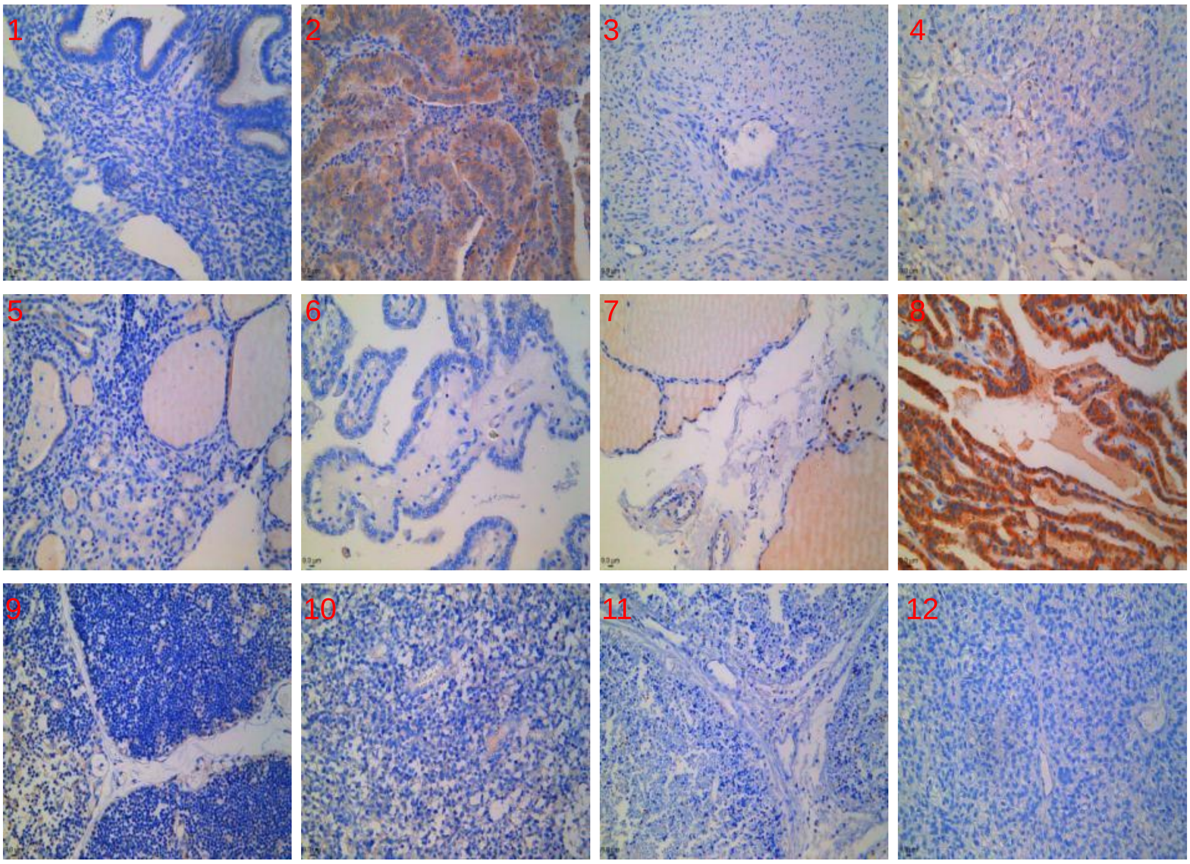

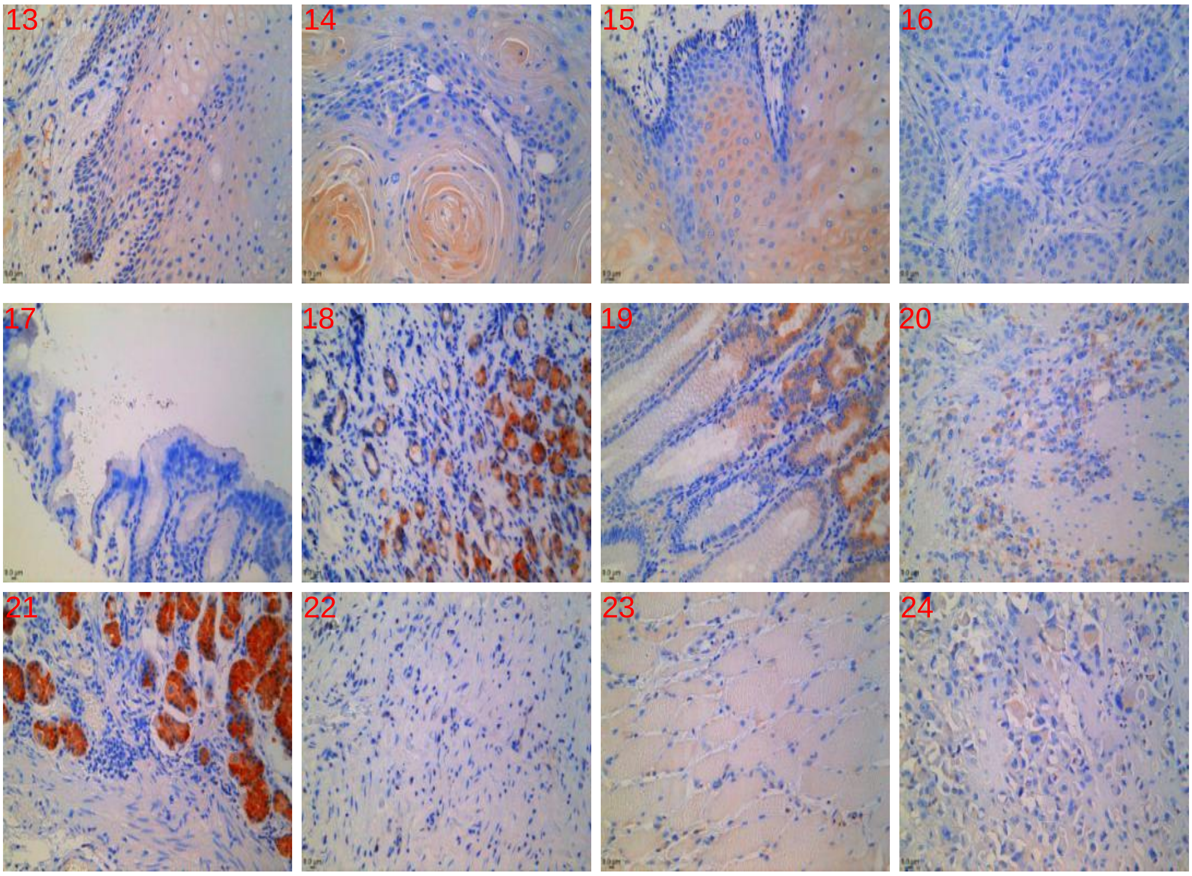
13
14
15
16
17
18
19
20
21
22
23
24

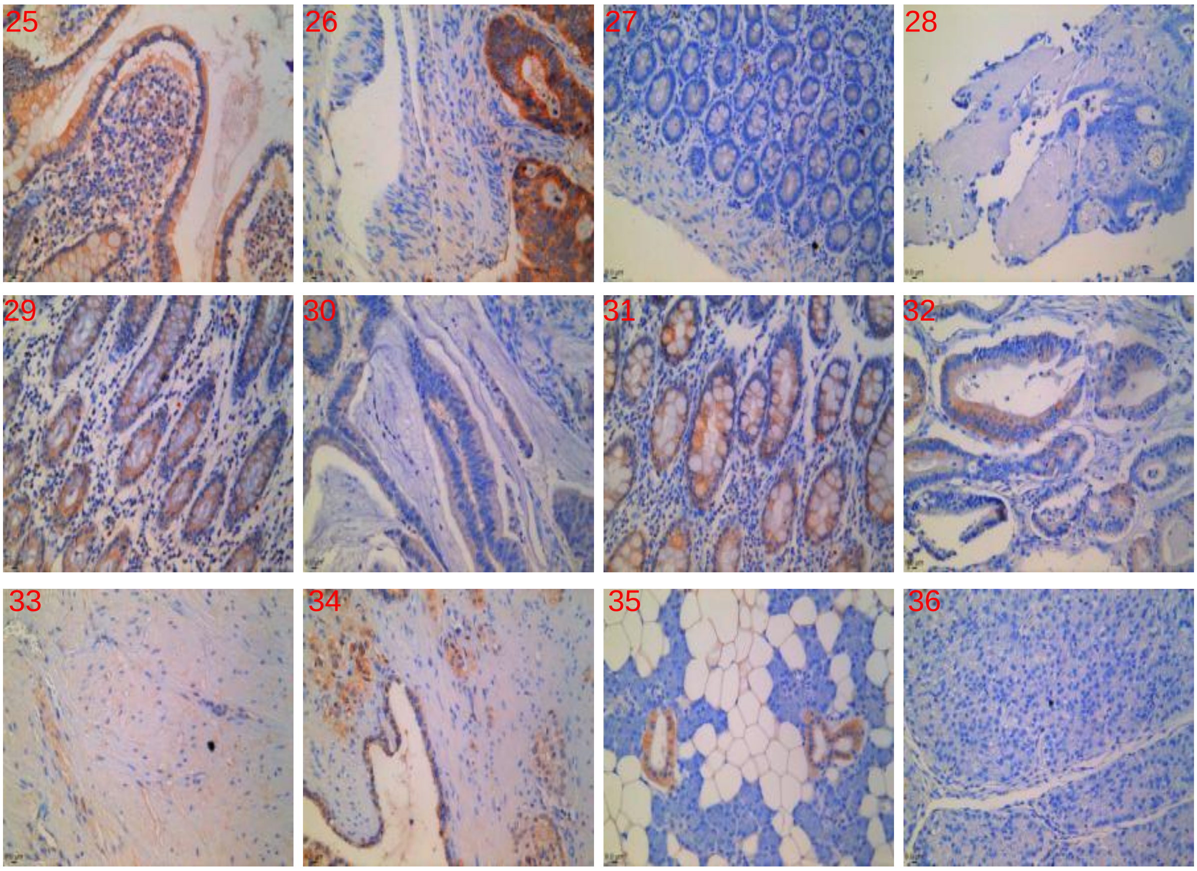
25
26
27
28
29
30
31
32
33
34
35
36

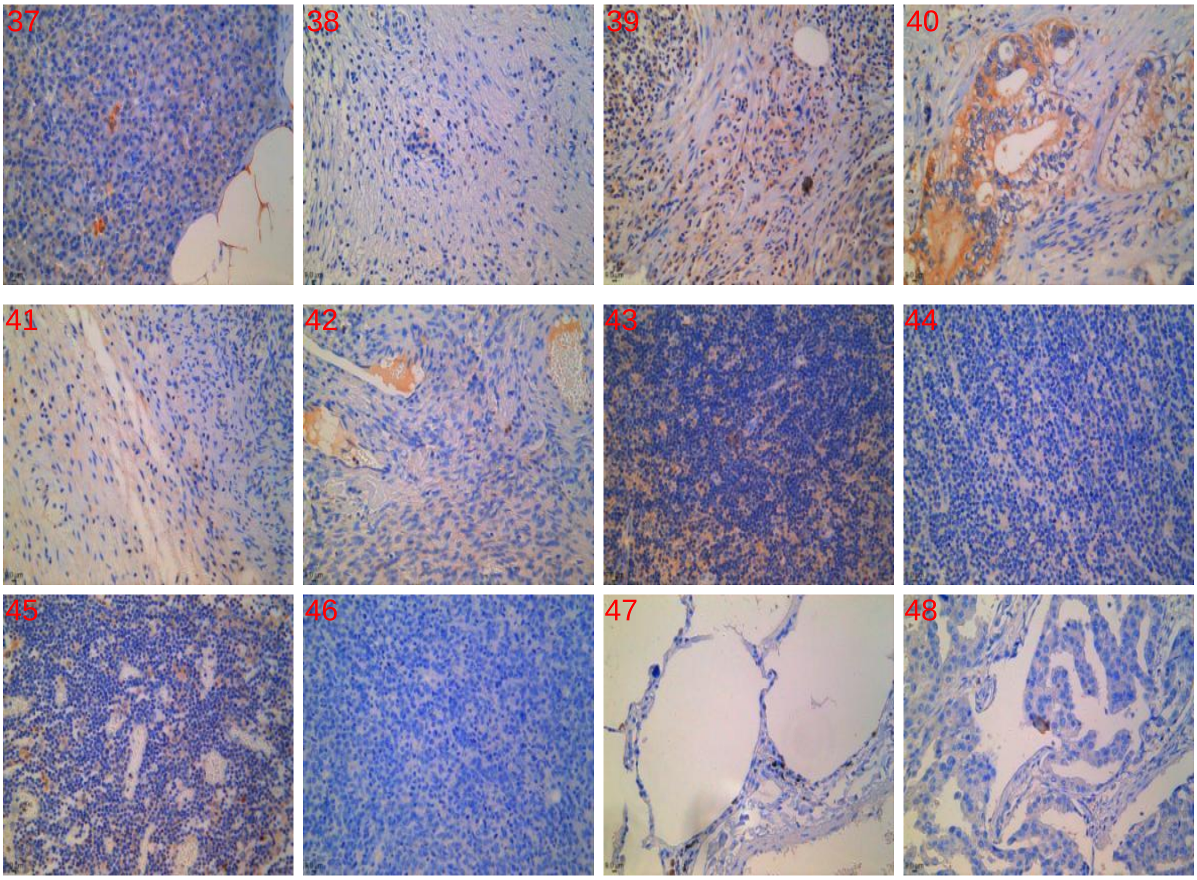
37
38
39
40
41
42
43
44
45
46
47
48

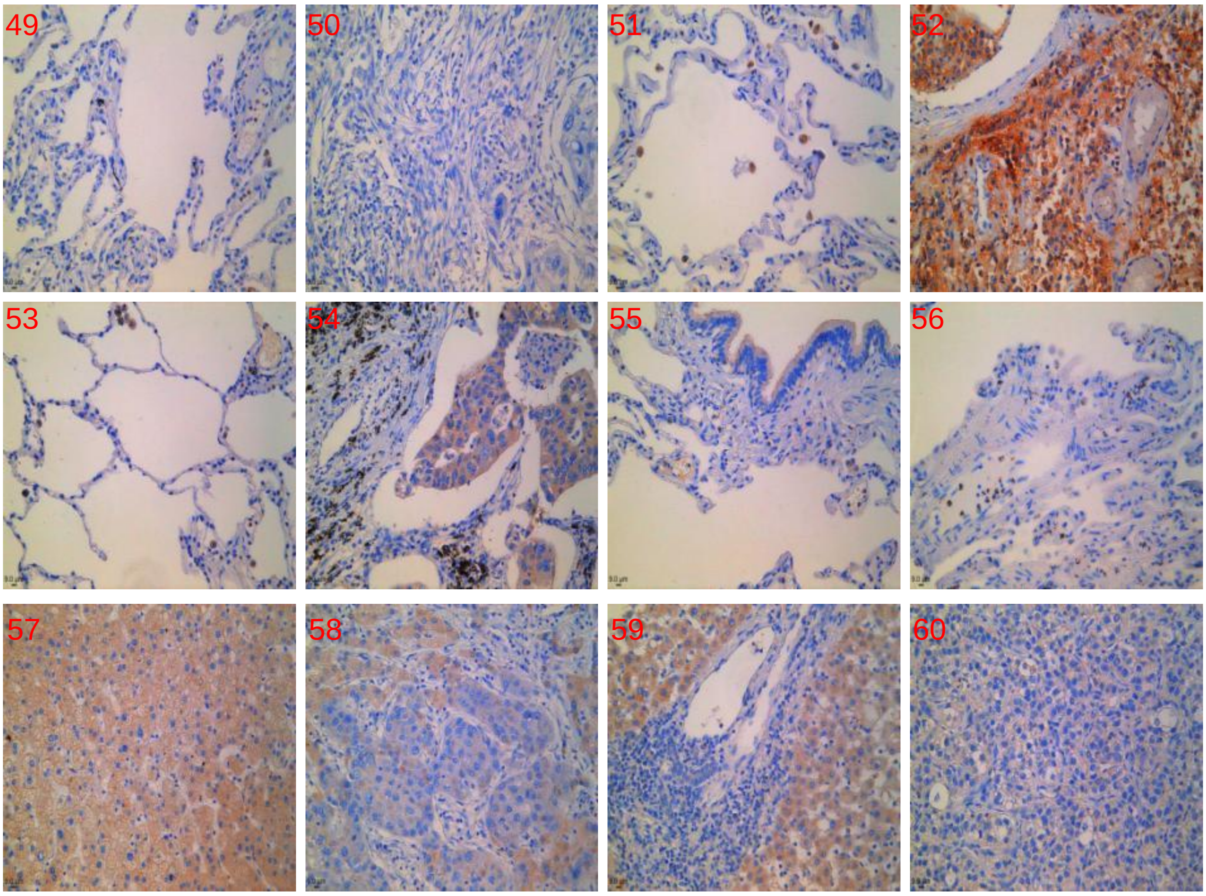
49
50
51
52
53
54
55
56
57
58
59
60

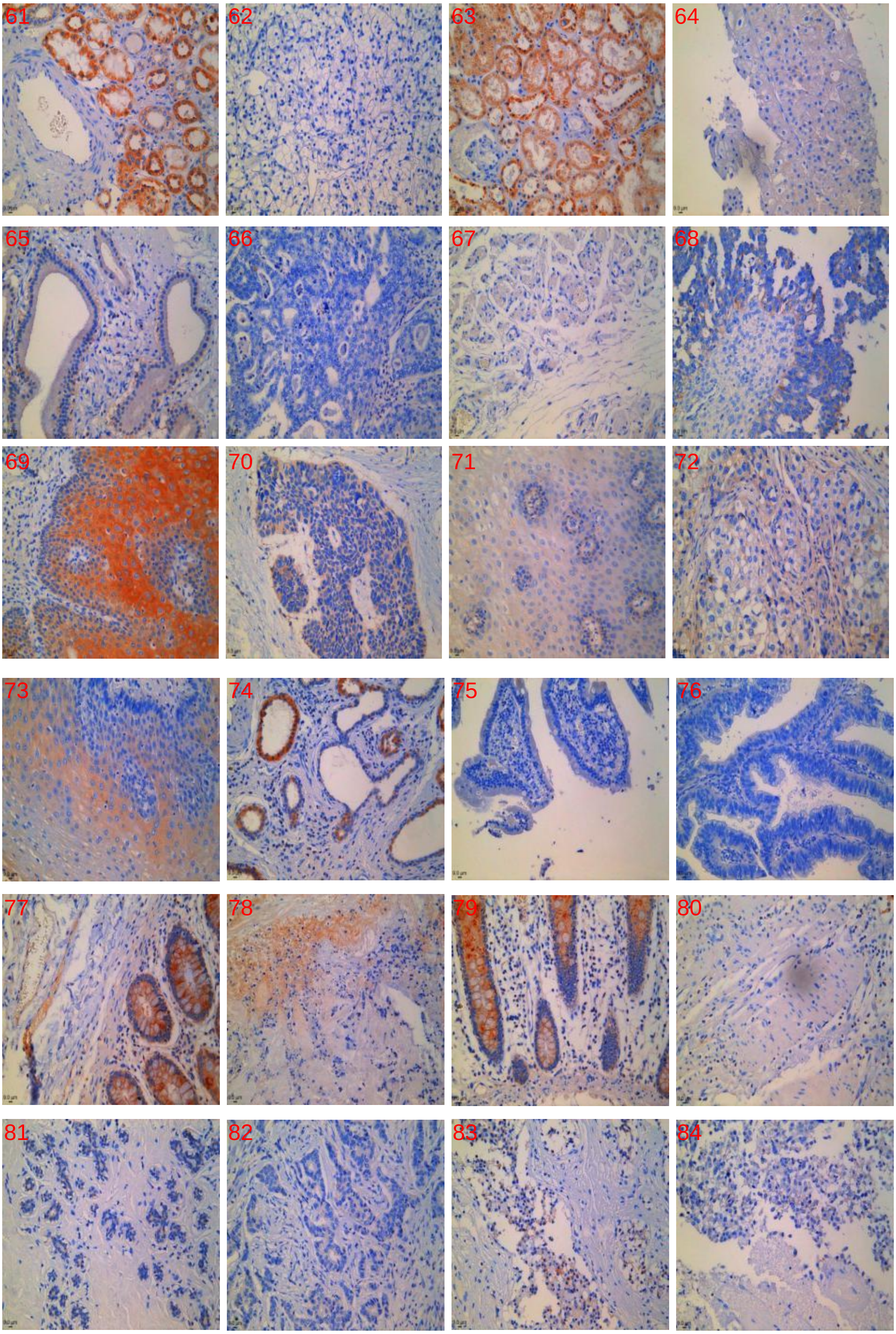
61
62
63
64
65
66
67
68
69
70
71
72
73
74
75
76
77
78
79
80
81
82
83
84

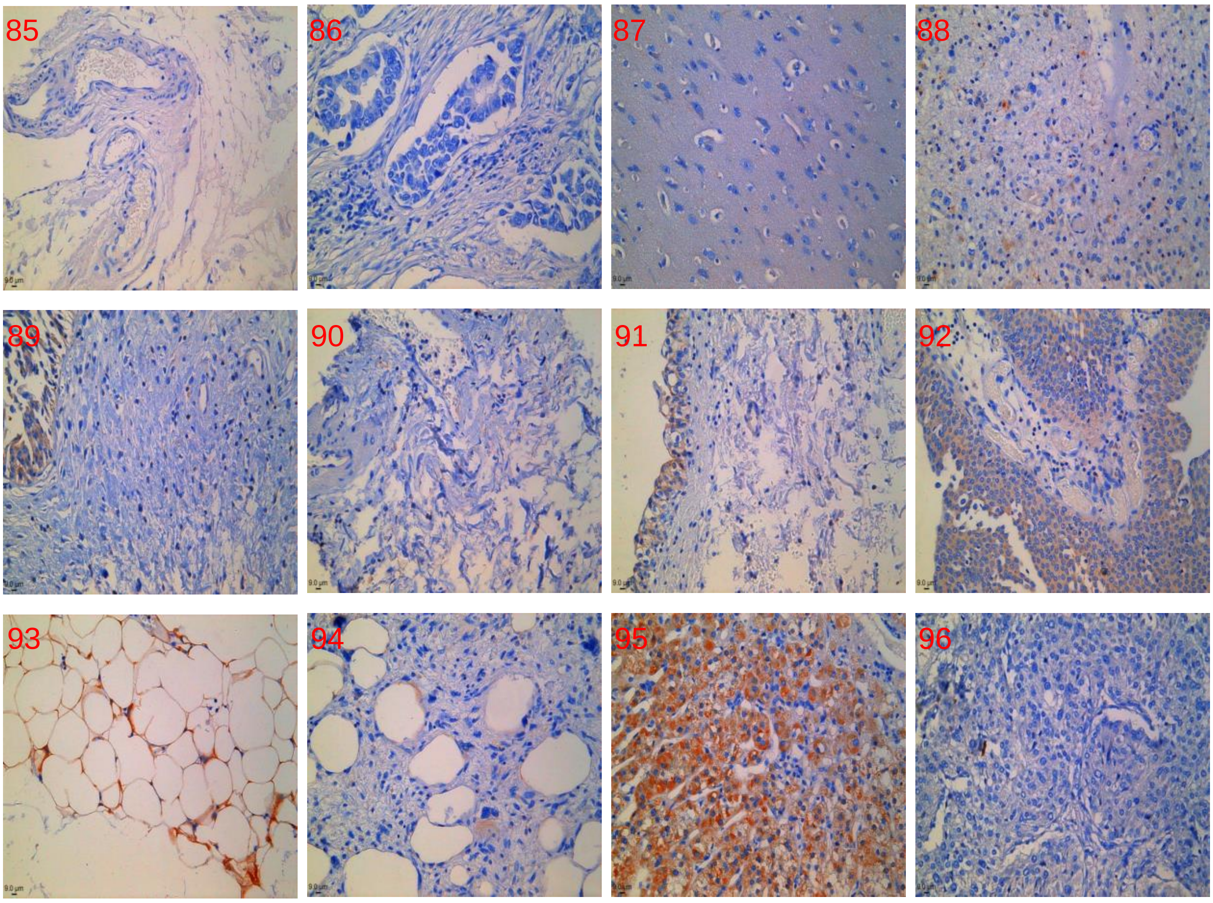
85
86
87
88
89
90
91
92
93
94
95
96
