# Supplementary material for: Nuclear Multidrug-Resistance Related Protein 1 Contributes to Multidrug-Resistance of Mucoepidermoid Carcinoma Mainly via Regulating Multidrug-Resistance Protein 1: A Human Mucoepidermoid Carcinoma Cells Model and Spearman's Rank Correlation Analysis
Source: PLoS One. 2013 Aug 27;8(8):e69611. doi: 10.1371/journal.pone.0069611 (PMC3754958; doi:10.1371/journal.pone.0069611)
Supplement: Materials and Methods S1 — Immunohistochemistry of the multiple tumor tissue arrays. (DOCX) [file pone.0069611.s002.docx]

**Supporting information 1**

**Materials and Methods S1:**

**Immunohistochemistry of the multiple tumor tissue arrays**

The multiple tumor tissue array section had 96 array cores in total which contained 48 different tumors and their corresponding normal tissues. The section thickness was 5μm and the array core diameter was 1mm. As described in the main text, immunohistochemistry assay was performed to examine the expression of MRP1 in the tissues. The slides were stained with 3,3’-diaminobenzidine (DAB) and counterstained with haematoxylin, then was observed under a microscope (Leica DMI6000 B Fully Automated Inverted Research Microscope, Germany) with 200×magnification.
