# Supplementary material for: Nuclear Multidrug-Resistance Related Protein 1 Contributes to Multidrug-Resistance of Mucoepidermoid Carcinoma Mainly via Regulating Multidrug-Resistance Protein 1: A Human Mucoepidermoid Carcinoma Cells Model and Spearman's Rank Correlation Analysis
Source: PLoS One. 2013 Aug 27;8(8):e69611. doi: 10.1371/journal.pone.0069611 (PMC3754958; doi:10.1371/journal.pone.0069611)
Supplement: Table S1 — Detailed information of tissue array panel. (DOCX) [file pone.0069611.s003.docx]

**Supporting information 3**

**Table S1: Detailed information of tissue array panel**

| Number | Age(Y) | Sex | Organ | Pathology |
| --- | --- | --- | --- | --- |
| 1 | 56 | F | Uterus | Cancer adjacent normal endometrial tissue |
| 2 | 70 | F | Uterus Tumor | Endometrial adenocarcinoma |
| 3 | 42 | F | Uterus | Cancer adjacent normal smooth muscle tissue |
| 4 | 35 | F | Uterus Tumor | Low-grade leiomyosarcoma |
| 5 | 30 | F | Thyroid | Cancer adjacent normal thyreoid tissue of No. 06 |
| 6 | 30 | F | Thyroid Tumor | Papillary carcinoma |
| 7 | 36 | F | Thyroid | Cancer adjacent normal thyreoid tissue of No. 08 |
| 8 | 36 | F | Thyroid Tumor | Papillary carcinoma |
| 9 | 0 | M | Thymus | Cancer adjacent normal thymic tissue |
| 10 | 38 | F | Thymus Tumor | Thymoma (type B) |
| 11 | 0. | M | Thymus | Cancer adjacent normal thymic tissue |
| 12 | 35 | M | Thymus Tumor | Thymoma (type C) |
| 13 | 35 | F | Tongue | Cancer adjacent normal glossal tissue of No. 14 |
| 14 | 35 | F | Tongue Tumor | Squamous cell carcinoma |
| 15 | 48 | F | Testis | Cancer adjacent normal glossal mucosa tissue of No. 16 |
| 16 | 48 | F | Testis Tumor | Squamous cell carcinoma |
| 17 | 62 | M | Stomach | Cancer adjacent normal gastric tissue of No. 18 |
| 18 | 62 | M | Stomach Tumor | Adenocarcinoma |
| 19 | 31 | M | Stomach | Cancer adjacent normal gastric tissue of No. 20 |
| 20 | 31 | M | Stomach Tumor | Adenocarcinoma |
| 21 | 32 | F | Stomach | Cancer adjacent normal gastric tissue of No. 22 |
| 22 | 32 | F | Stomach Tumor | Adenocarcinoma |
| 23 | 56 | M | Soft Tissue | Cancer adjacent normal skeletal muscle tissue of No. 24 |
| 24 | 56 | M | Soft Tissue Tumor | Rhabdomyosarcoma |
| 25 | 70 | M | Small intestine | Cancer adjacent normal small intestine tissue of No. 26 |
| 26 | 70 | M | Small intestine Tumor | Adenocarcinoma |
| 27 | 73 | M | Skin | Cancer adjacent normal small intestine tissue of No. 28 |
| 28 | 73 | M | Skin Tumor | Adenocarcinoma (sparse) |
| 29 | 55 | F | Rectum | Cancer adjacent normal rectal tissue of No. 30 |
| 30 | 55 | F | Rectum Tumor | Adenocarcinoma |
| 31 | 58 | F | Rectum | Cancer adjacent normal rectal tissue of No. 32 |
| 32 | 58 | F | Rectum Tumor | Adenocarcinoma |
| 33 | 21 | M | Prostate | Cancer adjacent normal prostatic tissue |
| 34 | 73 | M | Prostate Tumor | Adenocarcinoma |
| 35 | 49 | F | Parotid | Cancer adjacent normal parotid tissue of No. 36 |
| 36 | 49 | F | Parotid Tumor | Acinic cell carcinoma |
| 37 | 38 | M | Pancreas | Cancer adjacent normal pancreatic tissue of No. 38 |
| 38 | 38 | M | Pancreas Tumor | Adenocarcinoma (sparse) |
| 39 | 65 | F | Ovary | Cancer adjacent normal pancreatic tissue (pancreatitis) of No. 40 |
| 40 | 65 | F | Ovary Tumor | Adenocarcinoma |
| 41 | 68 | F | Ovary | Cancer adjacent normal ovarian tissue (sparse) of No. 42 |
| 42 | 68 | F | Ovary Tumor | Serous adenocarcinoma (ovarian tissue sparse) |
| 43 | 48 | F | Lymph Node | Cancer adjacent normal lymph node tissue |
| 44 | 13 | M | Lymphoma | Diffuse B-cell lymphoma |
| 45 | 79 | F | Lymph Node | Cancer adjacent normal lymph node tissue |
| 46 | 52 | M | Lymphoma | Diffuse large B-cell lymphoma |
| 47 | 42 | M | Lung | Cancer adjacent normal pulmonary tissue of No. 48 |
| 48 | 42 | M | Lung Tumor | Adenocarcinoma |
| 49 | 39 | M | Lung | Cancer adjacent normal pulmonary tissue of No. 50 |
| 50 | 39 | M | Lung Tumor | Adenocarcinoma |
| 51 | 60 | M | Lung | Cancer adjacent normal pulmonary tissue of No. 52 |
| 52 | 60 | M | Lung Tumor | Squamous cell carcinoma (sparse) |
| 53 | 53 | F | Lung | Cancer adjacent normal pulmonary tissue of No. 54 |
| 54 | 53 | F | Lung Tumor | Squamous cell carcinoma |
| 55 | 59 | F | Lung | Cancer adjacent normal pulmonary tissue of No. 56 |
| 56 | 59 | F | Lung Tumor | Squamous cell carcinoma (pulmonary tissue) |
| 57 | 65 | F | Liver | Cancer adjacent normal hepatic tissue (virus hepatitis) of No. 58 |
| 58 | 65 | F | Liver Tumor | Hepatocellullar carcinoma |
| 59 | 48 | M | Liver | Cancer adjacent normal hepatic tissue (with fatty degeneration) of No. 60 |
| 60 | 48 | M | Liver Tumor | Hepatocellullar carcinoma |
| 61 | 54 | M | Kidney | Cancer adjacent normal renal tubule tissue of No. 62 |
| 62 | 54 | M | Kidney Tumor | Clear cell carcinoma |
| 63 | 57 | F | Kidney | Cancer adjacent normal renal tissue of No. 64 |
| 64 | 57 | F | Kidney Tumor | Granular cell carcinoma |
| 65 | 35 | F | Gall Bladder | Cancer adjacent normal cholecystic tissue of No. 66 |
| 66 | 35 | F | Gall Bladder Tumor | Adenocarcinoma |
| 67 | 49 | F | Fallopian Tube | Cancer adjacent normal fallopian tube tissue of No. 68 |
| 68 | 49 | F | Fallopian Tube Tumor | Adenocarcinoma |
| 69 | 52 | M | Esophagus | Cancer adjacent normal esophageal tissue of No. 70 |
| 70 | 52 | M | Esophagus Tumor | Squamous cell carcinoma |
| 71 | 56 | M | Esophagus | Cancer adjacent normal esophageal tissue of No. 72 |
| 72 | 56 | M | Esophagus Tumor | Squamous cell carcinoma |
| 73 | 53 | M | Esophagus | Cancer adjacent normal esophageal mucosa tissue |
| 74 | 64 | M | Esophagus Tumor | Mucinous adenocarcinoma (sparse) |
| 75 | 51 | M | Duodenum | Cancer adjacent normal duodenal tissue of No. 76 |
| 76 | 51 | M | Duodenum Tumor | Papillary adenocarcinoma |
| 77 | 69 | M | Colon | Cancer adjacent normal colonic tissue of No. 78 |
| 78 | 69 | M | Colon Tumor | Mucinous adenocarcinoma (sparse) |
| 79 | 42 | F | Colon | Cancer adjacent normal colonic tissue of No. 80 |
| 80 | 42 | F | Colon Tumor | Adenocarcinoma (smooth muscle) |
| 81 | 44 | F | Breast | Cancer adjacent normal breast tissue of No. 82 |
| 82 | 44 | F | Breast Tumor | Infiltrating ductal carcinoma |
| 83 | 45 | F | Breast | Cancer adjacent normal breast tissue of No. 84 |
| 84 | 45 | F | Breast Tumor | Infiltrating ductal carcinoma |
| 85 | 40 | F | Breast | Cancer adjacent normal breast tissue of No. 86 |
| 86 | 40 | F | Breast Tumor | Infiltrating ductal carcinoma |
| 87 | 30 | F | Brain | Cancer adjacent normal ectocinerea tissue of No. 88 |
| 88 | 30 | F | Brain Tumor | Astrocytoma |
| 89 | 70 | M | Bladder | Cancer adjacent normal urocystic tissue of No. 90 |
| 90 | 70 | M | Bladder Tumor | Transitional cell carcinoma (fibrous tissue) |
| 91 | 62 | M | Bladder | Cancer adjacent normal urocystic tissue (fibrous tissue) of No. 92 |
| 92 | 62 | M | Bladder Tumor | Transitional cell carcinoma |
| 93 | 73 | M | Adipose | Cancer adjacent normal epoploon tissue |
| 94 | 46 | M | Adipose Tumor | Pleomorphic liposarcoma |
| 95 | 46 | F | Adrenal | Cancer adjacent normal adrenal tissue |
| 96 | 34 | F | Adrenal Tumor | Pheochromocytoma |

*Y, year; F, female; M, male
